# Local Distributions of Wealth to Describe Health Inequalities in India: A New Approach for Analyzing Nationally Representative Household Survey Data, 1992–2008

**DOI:** 10.1371/journal.pone.0110694

**Published:** 2014-10-30

**Authors:** Diego G. Bassani, Daniel J. Corsi, Michelle F. Gaffey, Aluisio J. D. Barros

**Affiliations:** 1 Department of Paediatrics, University of Toronto, Toronto, Ontario, Canada; 2 Harvard Center for Population and Development Studies, Harvard University, Cambridge, Massachusetts, United States of America; 3 Centre for Global Child Health, The Hospital for Sick Children, Toronto, Ontario, Canada; 4 Postgraduate Program in Epidemiology, Federal University of Pelotas, Pelotas, Brazil; Örebro University, Sweden

## Abstract

**Background:**

Worse health outcomes including higher morbidity and mortality are most often observed among the poorest fractions of a population. In this paper we present and validate national, regional and state-level distributions of national wealth index scores, for urban and rural populations, derived from household asset data collected in six survey rounds in India between 1992–3 and 2007–8. These new indices and their sub-national distributions allow for comparative analyses of a standardized measure of wealth across time and at various levels of population aggregation in India.

**Methods:**

Indices were derived through principal components analysis (PCA) performed using standardized variables from a correlation matrix to minimize differences in variance. Valid and simple indices were constructed with the minimum number of assets needed to produce scores with enough variability to allow definition of unique decile cut-off points in each urban and rural area of all states.

**Results:**

For all indices, the first PCA components explained between 36% and 43% of the variance in household assets. Using sub-national distributions of national wealth index scores, mean height-for-age z-scores increased from the poorest to the richest wealth quintiles for all surveys, and stunting prevalence was higher among the poorest and lower among the wealthiest. Urban and rural decile cut-off values for India, for the six regions and for the 24 major states revealed large variability in wealth by geographical area and level, and rural wealth score gaps exceeded those observed in urban areas.

**Conclusions:**

The large variability in sub-national distributions of national wealth index scores indicates the importance of accounting for such variation when constructing wealth indices and deriving score distribution cut-off points. Such an approach allows for proper within-sample economic classification, resulting in scores that are valid indicators of wealth and correlate well with health outcomes, and enables wealth-related analyses at whichever geographical area and level may be most informative for policy-making processes.

## Background

Worse health outcomes including higher morbidity and mortality are most often observed among the poorest fractions of the population [1]. This is in part due to lower health service use, more limited access to health interventions and poorer nutritional status [1], [2], but health inequalities are a consequence of complex processes including multidimensional drivers reflecting differences in economic status and social characteristics such as gender and ethnicity. The growing need to better understand the influence of poverty on health has dramatically increased the interest in research [3], [4] and also the programmatic attention on health inequalities in low- and middle-income countries [2], [5], [6], [7].

The development of new methods for estimating household economic status has facilitated new research on the effects of wealth disparities on health [8], [9]. Preferred measures of economic status require data on household income or consumption, but these indicators are hard to define in some settings, difficult to collect on a large scale and prone to misclassification [10]. Conversely, data on ownership of durable goods, housing characteristics and access to infrastructure are easier to measure and commonly available from household surveys, and these can be used compositely to classify households’ relative wealth [11]. As such, asset-based wealth indices derived through principal components analysis are increasingly being used to characterize economic status in household survey analyses of health inequalities [12], [13] In addition, such surveys are often repeated periodically in a given population, allowing indices to be updated as needed to ensure the most relevant assets are included.

While asset-based wealth indices are typically constructed at the national level, the use of national wealth score distributions for sub-national analyses is problematic [14], [15], [16]. For example, ignoring the wealth score distribution at the geographic level of interest (e.g. district, state, or region) may result in a large proportion of one population (e.g. state) being assigned to the top or bottom of the wealth distribution of another population (e.g. region), thereby hiding level-specific wealth gradients. The use of geographical-level wealth distributions allows one to correctly classify households according to the most appropriate wealth score distribution, enabling proper comparisons across different states, regions or countries and across different geographical levels.

In India, due to the large, socio-economically diverse population and the decentralized decision-making and policy-setting structures, the use of wealth distributions at multiple geographic levels is especially important for analyzing and addressing health inequalities. However, while national and sub-national wealth distributions in India have been devised and employed previously [17], [18], [19], a comprehensive set of wealth distributions at multiple geographic levels in India has not been made available in the literature before now. In this paper we present national, regional and state-level distributions of national wealth index scores, for urban and rural populations separately, derived from household asset data collected in the three rounds of the Demographic and Health Survey, known as the National Family Health Survey (NFHS) in India [17], [18], [20], and in three rounds of the District Level Household Survey (DLHS) [21], [22], [23]. The six surveys cover a period between 1992–3 and 2007–8 and allow for a standardized measure of wealth that can be used in survey-specific analyses as well as for comparisons across surveys/time-points. We validate our indices by analyzing height-for-age as one example of a health inequality which has previously been shown to have marked differences by wealth quintile [24]. Further, we illustrate the important misclassification of households that may result from sub-national analyses that use national wealth distributions. We propose that the urban and rural wealth score decile cut-off values that we present for different geographical levels can be used to improve future analyses of health inequalities in India and ultimately inform the decentralized policy-making processes by which such inequalities can be effectively addressed.

## Methods

### Ethics statement

This secondary analysis of anonymized survey data available in the public domain did not require prior approval from an ethics review board. The original surveys received approval by the relevant ethics review boards.

### Data

The National Family Health Survey (NFHS) is a large-scale, nationally representative survey of Indian households providing state- and national-level estimates of key demographic and health indicators. Three rounds of the survey have been conducted to date (NFHS-1 in 1992–3, NFHS-2 in 1998–9 and NFHS-3 in 2005–6), each using an equivalent multi-stage sampling approach and including more than 85,000 households, with an overall response rate above 98%. Sampling design, sample size and response rate details are published in the round-specific survey reports [17], [18], [20]. In addition to demographic and health information, the NFHS collects data on household socioeconomic characteristics, including ownership of various assets, housing construction materials, and access to electricity. The assets included in the survey questionnaire varies between rounds.

The District Level Household Survey (DLHS) has been conducted in four rounds to date: DLHS-1 in 1998–9, DLHS-2 in 2002–4 DLHS-3 in 2007–8 and DLHS-4 in 2012–13. This survey collects information similar to the NFHS surveys but uses a sampling frame tailored to be representative at the district level [21], [22], [23]. Here we use data from the first three DLHS rounds, as datasets from the most recent round are not yet available in the public domain.

### Wealth indices

We initially constructed separate indices for urban and rural setting in each survey, using different lists of assets. While there are fundamental differences in infrastructure and lifestyle between urban and rural areas, our comparison of the separate indices to a single national index revealed that the national index performed as well as the separate urban and rural indices in all states, with the advantage of being simpler to develop and implement in future research. However, because the assets on which data were collected through the surveys differed over time, a separate national index was constructed for each of the six surveys.

We derived our indices through principal components analysis (PCA) using Stata 12 [25]. PCA is a multivariate statistical technique for reducing a larger number of variables to a smaller number of dimensions [26]. PCA can summarize the variance of different types of variables with no specific distribution, generating a score that captures, in its first component, the greatest amount of data variability explained by one linear combination of variables. This approach is well-suited for handling the mixture of discrete and continuous data typically collected in household surveys [13]. The use of variables measured on different scales can result in different variances and this may produce quite different results in the PCA depending on whether one uses covariance or correlation matrices for the calculations. Large variances will dominate the first principal component if covariance matrices are used. For this reason, the PCA was performed using standardized variables from a correlation matrix, which minimizes the differences in variance. To generate valid indices that were as simple as possible, each index was constructed with the minimum number of variables/assets that would produce scores with enough variability to allow us to define unique cut-off points for each urban and rural area of all states.

The indices include 16 assets for NFHS-3 (2005–6), 14 assets for NFHS-2 (1998–9) and 11 assets for NFHS-1 (1992–3). The index for DLHS-3 (2007–8) includes 14 assets, the index for DLHS-2 (2002–4) includes 10 assets and the index for DLHS-1 (1998–9) includes 9 assets (Tables 1–6). Binary coding (i.e. yes/no) was applied to all but two assets; highest education level achieved by the household head was categorized as none/primary/secondary/higher than secondary, while the number of bedrooms in the dwelling was categorized as one/two/three/four or more (thereby ensuring that at least 5% of households were included in the highest category).

**Table 1:** Assets selected to create a national wealth index from the NFHS-3 (2005–6) survey, with coding definitions.

Variable	Coding	Loading	SD	Coefficient
**Common assets**				
Highest educationlevel attained(head of the household)	0 = No education1 = Primary2 = Secondary3 = Higher than secondary	0.248	1.028	24
Refrigerator	1 = Yes 0 = No	0.270	0.360	75
Scooter/Motorcycle	1 = Yes 0 = No	0.253	0.378	67
Telephone	1 = Yes 0 = No	0.253	0.348	73
Electric Fan	1 = Yes 0 = No	0.243	0.467	52
Pressure Cooker	1 = Yes 0 = No	0.310	0.485	64
Chair	1 = Yes 0 = No	0.283	0.498	57
Table	1 = Yes 0 = No	0.298	0.496	60
Sewing Machine	1 = Yes 0 = No	0.219	0.389	56
Number of bedrooms	1 = 1 2 = 2 3 = 3 4 = 4+	0.159	0.816	20
Mobile-phone	1 = Yes 0 = No	0.259	0.374	69
Mattress	1 = Yes 0 = No	0.234	0.494	47
Electricity	1 = Yes 0 = No	0.243	0.467	52
Television	1 = Yes 0 = No	0.302	0.497	61
Radio	1 = Yes 0 = No	0.161	0.462	35
Bed	1 = Yes 0 = No	0.131	0.377	35
		*rho = 38.5*		

SD: Standard Deviation.

**Table 2:** Assets selected to create a national wealth index from the NFHS-2 (1998–9) survey, with coding definitions.

Variable	Coding	Loading	SD	Coefficient
**Common assets**				
Highest educationlevel attained(head of the household)	0 = No education1 = Primary2 = Secondary3 = Higher than secondary	0.250	1.062	24
Refrigerator	1 = Yes 0 = No	0.264	0.308	86
Scooter/Motorcycle	1 = Yes 0 = No	0.245	0.316	78
Telephone	1 = Yes 0 = No	0.231	0.262	88
Electric Fan	1 = Yes 0 = No	0.309	0.498	62
Pressure Cooker	1 = Yes 0 = No	0.314	0.456	69
Chair	1 = Yes 0 = No	0.294	0.498	59
Table	1 = Yes 0 = No	0.300	0.489	61
Sewing Machine	1 = Yes 0 = No	0.235	0.387	61
Number of Rooms	1 = 1 2 = 2 3 = 3 4 = 4+	0.214	1.114	19
Mattress	1 = Yes 0 = No	0.275	0.499	55
Electricity	1 = Yes 0 = No	0.253	0.490	52
Television	1 = Yes 0 = No	0.316	0.473	67
Radio	1 = Yes 0 = No	0.210	0.485	43
		*rho = 43.1*		

SD: Standard Deviation.

**Table 3:** Assets selected to create a national wealth index from the NFHS-1 (1992–3) survey, with coding definitions.

Variable	Coding	Loading	SD	Coefficient
**Common assets**				
Highest educationlevel attained(head of the household)	0 = No education1 = Primary2 = Secondary3 = Higher than secondary	0.299	0.968	31
Refrigerator	1 = Yes 0 = No	0.292	0.251	116
Scooter/Motorcycle	1 = Yes 0 = No	0.276	0.273	101
Electric Fan	1 = Yes 0 = No	0.361	0.468	77
Sofa set	1 = Yes 0 = No	0.303	0.290	104
Clock	1 = Yes 0 = No	0.301	0.500	60
Sewing Machine	1 = Yes 0 = No	0.280	0.384	73
Number of Rooms	1 = 1 2 = 2 3 = 3 4 = 4+	0.210	1.108	19
Electricity	1 = Yes 0 = No	0.294	0.500	59
Television	1 = Yes 0 = No	0.360	0.406	89
Radio	1 = Yes 0 = No	0.280	0.488	57
		*rho = 40.0*		

SD: Standard Deviation.

**Table 4:** Assets selected to create a national wealth index from the DLHS-3 (2007–8) survey, with coding definitions.

Variable	Coding	Loading	SD	Coefficient
**Common assets**				
Highest educationlevel attained(head of the household)	0 = No education1 = Primary2 = Secondary3 = Higher than secondary	0.238	1.060	22
Refrigerator	1 = Yes 0 = No	0.285	0.330	86
Scooter/Motorcycle	1 = Yes 0 = No	0.261	0.367	71
Telephone	1 = Yes 0 = No	0.226	0.301	75
Electric Fan	1 = Yes 0 = No	0.297	0.497	60
Pressure Cooker	1 = Yes 0 = No	0.319	0.477	67
Chair	1 = Yes 0 = No	0.284	0.499	57
Table	1 = Yes 0 = No	0.296	0.492	60
Sewing Machine	1 = Yes 0 = No	0.238	0.377	63
Number of bedrooms	1 = 1 2 = 2 3 = 3 4 = 4+	0.174	0.888	20
Mobile-phone	1 = Yes 0 = No	0.304	0.474	64
Mattress	1 = Yes 0 = No	0.200	0.492	41
Electricity	1 = Yes 0 = No	0.254	0.473	54
Television	1 = Yes 0 = No	0.320	0.451	71
		*rho = 39.8*		

SD: Standard Deviation.

**Table 5:** Assets selected to create a national wealth index from the DLHS-2 (2002–4) survey, with coding definitions.

Variable	Coding	Loading	SD	Coefficient
**Common assets**				
Highest educationlevel attained(head of the household)	0 = No education1 = Primary2 = Secondary3 = Higher than secondary	0.320	1.124	28
Scooter/Motorcycle	1 = Yes 0 = No	0.333	0.368	91
Telephone	1 = Yes 0 = No	0.351	0.373	94
Electric Fan	1 = Yes 0 = No	0.342	0.467	73
Car	1 = Yes 0 = No	0.193	0.175	111
Bicycle	1 = Yes 0 = No	0.130	0.499	26
Sewing Machine	1 = Yes 0 = No	0.317	0.421	75
Electricity	1 = Yes 0 = No	0.342	0.467	73
Television	1 = Yes 0 = No	0.414	0.496	83
Radio	1 = Yes 0 = No	0.250	0.480	52
		*rho = 36.0*		

SD: Standard Deviation.

**Table 6:** Assets selected to create a national wealth index from the DLHS-1 (1998–9) survey, with coding definitions.

Variable	Coding	Loading	SD	Coefficient
**Common assets**				
Highest educationlevel attained(head of the household)	0 = No education1 = Primary2 = Secondary3 = Higher than secondary	0.332	1.117	30
Car	1 = Yes 0 = No	0.153	0.161	95
Scooter/Motorcycle	1 = Yes 0 = No	0.313	0.330	95
Electric Fan	1 = Yes 0 = No	0.440	0.499	88
Bicycle	1 = Yes 0 = No	0.176	0.500	35
Sewing Machine	1 = Yes 0 = No	0.339	0.423	80
Electricity	1 = Yes 0 = No	0.379	0.486	78
Television	1 = Yes 0 = No	0.438	0.484	90
Radio	1 = Yes 0 = No	0.306	0.491	62
		*rho = 37.0*		

SD: Standard Deviation.

An index coefficient *c* for each asset was calculated using the expression 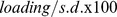 rounded to the nearest integer. The wealth scores for each household were then calculated using the expression 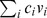 where *c* ***_i_*** represents the index coefficient and *v* ***_i_*** the coded value of the *i*th asset.

From the resulting score assigned to each household, the national, regional and state score distributions were derived for each survey round, for urban and rural areas separately, and the score value for each stratum-specific decile was then identified. To account for the complex survey design, the sampling weights provided with the survey datasets were used for all analyses.

## Results

Tables 1–6 give the indexed variables for each survey respectively, with their factor loadings, standard deviations and index coefficients. For the NFHS-3 (Table 1), the first component explained 38.5% of the data variability. The first component explained 43.1% of the variability in the NFHS-2 data (Table 2), and 40.0% of the variability in the NFHS-1 data (Table 3). For the DLHS surveys, the first component explained 39.8% of the data variability in DLHS-3 (Table 4), 36.0% of the variability in the DLHS-2 data (Table 5), and 37% of the variability in DLHS-1 (Table 6).

Using the 2006 WHO growth standards [27] we analyzed the distribution of the mean height-for-age z-scores across wealth quintiles defined by local reference cut-off points. As expected, the mean height-for-age z-score increased from the poorest to the richest wealth quintiles, and similarly, prevalence of stunting was higher among the poorest and lower among the wealthiest. These trends were consistent for all three rounds of the NFHS. We calculated the Pearson correlation between the continuous wealth score and height-for-age z-score for all children under age 5. The values were 0.23 (p-value<0.0001) in urban areas and 0.18 (p-value<0.0001) in rural areas (NFHS-3). Spearman rank correlations had very similar results: 0.27 in urban areas and 0.20 in rural areas (p-value<0.001). These values were similar to correlations obtained between height-for-age z-score and the originally constructed NFHS-3 wealth index based on the DHS methodology [28]. The Pearson correlations were 0.25 and 0.19 (p-value<0.001) and the Spearman rank correlations were 0.28 and 0.21 (p-value<0.001) in urban and rural areas, respectively.

**Figure 1:**
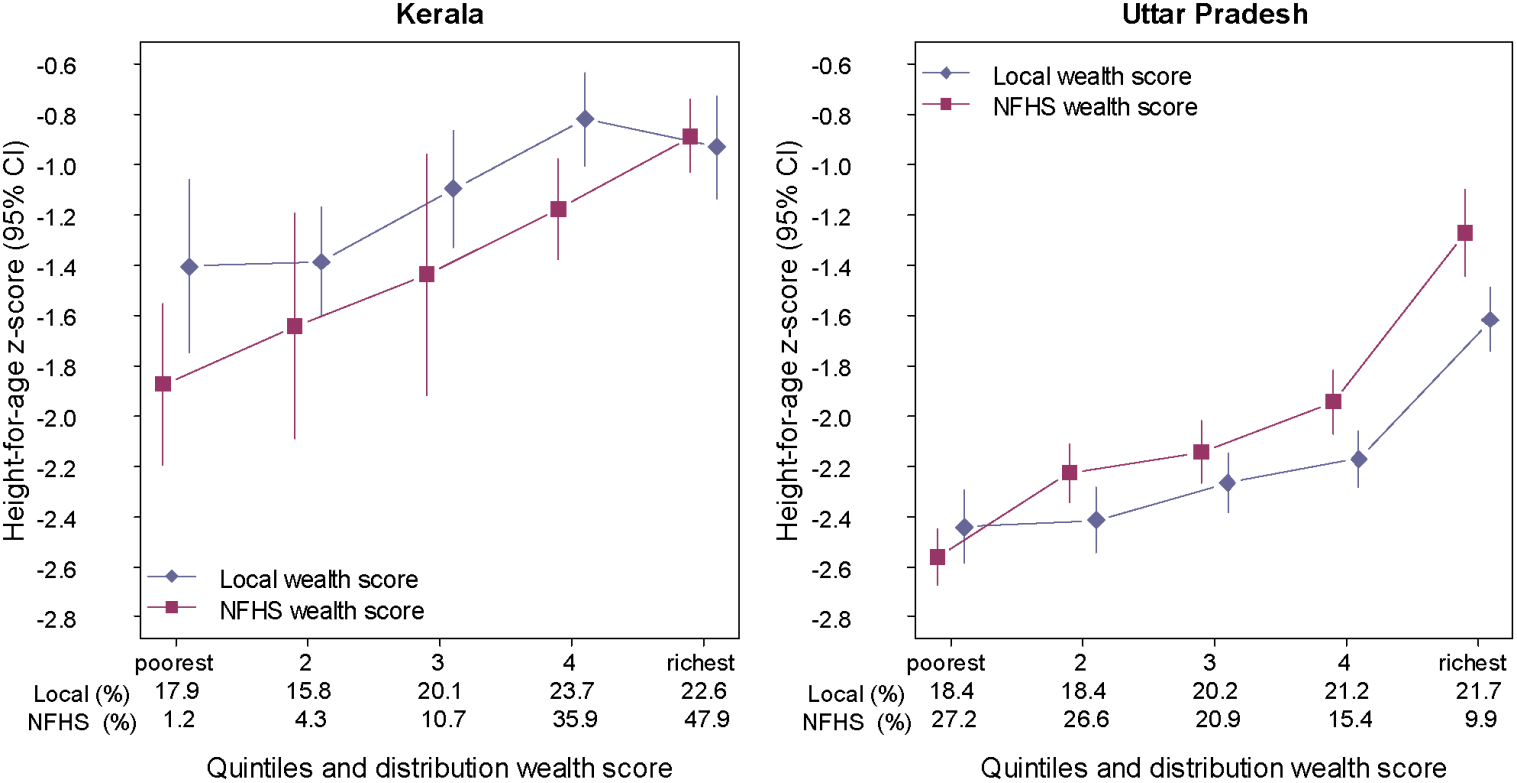
Mean height-for-age by wealth score quintiles derived from local state and urban/rural cut points and from NFHS national cut points in the states of Kerala and Uttar Pradesh, 2005–6.

**Figure 2:**
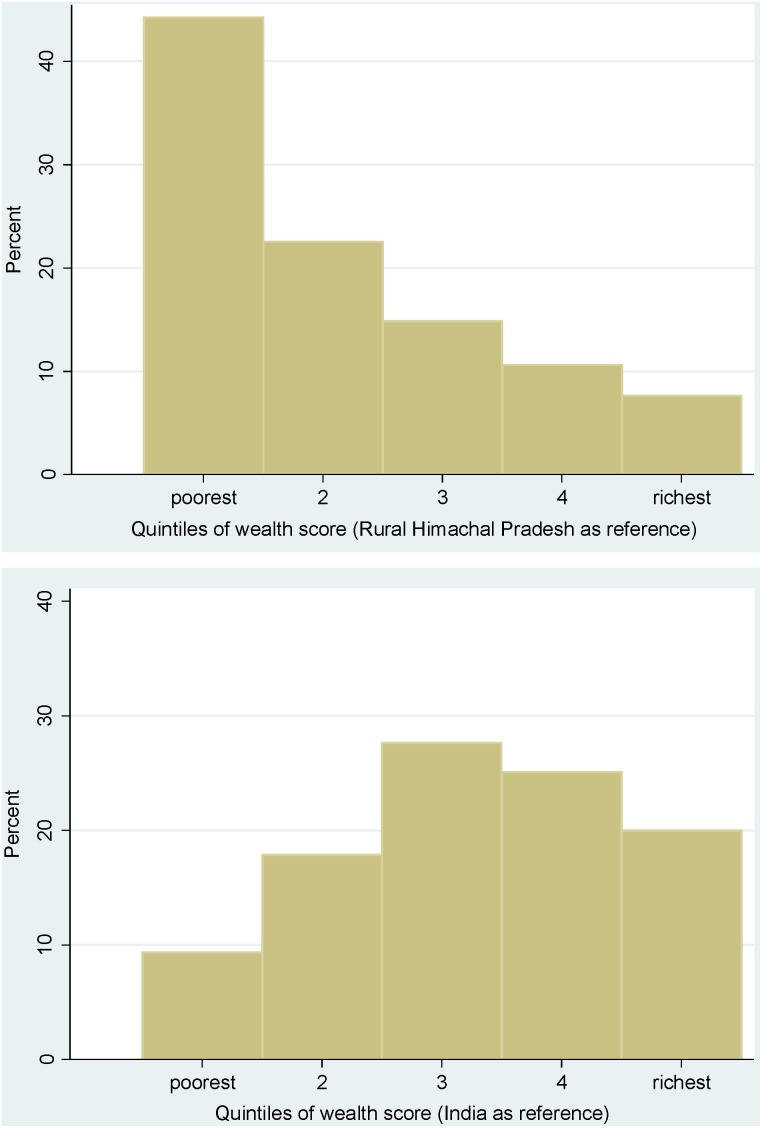
Prevalence of stunting by wealth score quintiles derived from local state and urban/rural cut points and from NFHS national cut points in the states of Kerala and Uttar Pradesh, 2005–6.

In Figures 1 and 2 we present state-level analyses for Kerala and Uttar Pradesh in 2005–6 showing mean height-for-age z-score (Figure 1) and stunting prevalence (Figure 2) by wealth quintile, and comparing estimates for locally defined quintiles with estimates for the national quintiles originally defined in the NFHS-3. Kerala and Uttar Pradesh were chosen to represent the diverse levels of economic development and health indicators. Kerala is among the richest states in India and ranks highest in terms of conventional measures of health and economic development, while Uttar Pradesh is one of the poorest states and ranks among the lowest by infant mortality rate, literacy, and per capita income [18], [29], [30]. Based on the original NFHS-3 national quintiles, nearly 50% of children with survey height measurements in Kerala are classified in the richest quintile, whereas local cut-offs result in a much more even distribution of children across quintiles. The wealth gradient for child linear growth in Kerala appears steeper when the national quintiles are used compared to the locally defined quintiles. The strength of this relationship is likely overstated because, with fewer individuals classified in the poorest quintiles based on the national cut-offs, there is additional uncertainty in estimating the mean height-for-age in these groups. This exaggeration of the state-specific wealth gradient when using national quintiles is similarly shown in Uttar Pradesh, where only 10% of children were classified in the richest national quintile.

**Figure 3:**
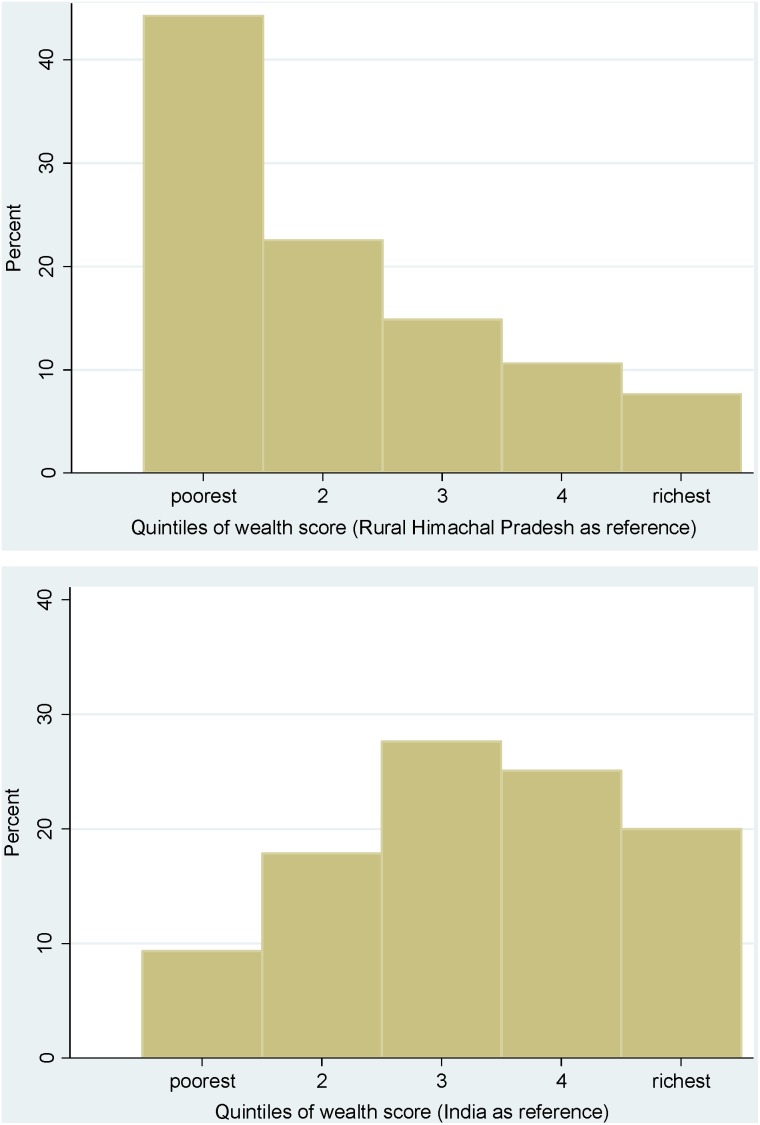
Distribution of wealth scores from a subsample of the rural Himachal Pradesh population by reference wealth quintiles for the rural state (top) and for rural India (bottom) in 2005–06 (NFHS-3).

For analyzing health inequalities, the importance of using reference distributions from the most appropriate geographical level is further illustrated in Figure 3. We compare the wealth score distributions of a sub-sample of eight rural villages in Himachal Pradesh with the full rural distribution for Himachal Pradesh (top panel) and with the rural distribution for all of India (bottom panel). If the sub-sampled villages had a similar wealth distribution to that of the state, all bars in the upper histogram (representing each quintile) would include approximately 20% of the sub-sampled village households. However, the sub-sample distribution is in fact largely skewed towards the lowest state-specific wealth quintile. Alternately, when compared to the rural wealth distribution of the whole country the sub-sample distribution is skewed to the higher national quintiles.

**Figure 4:**
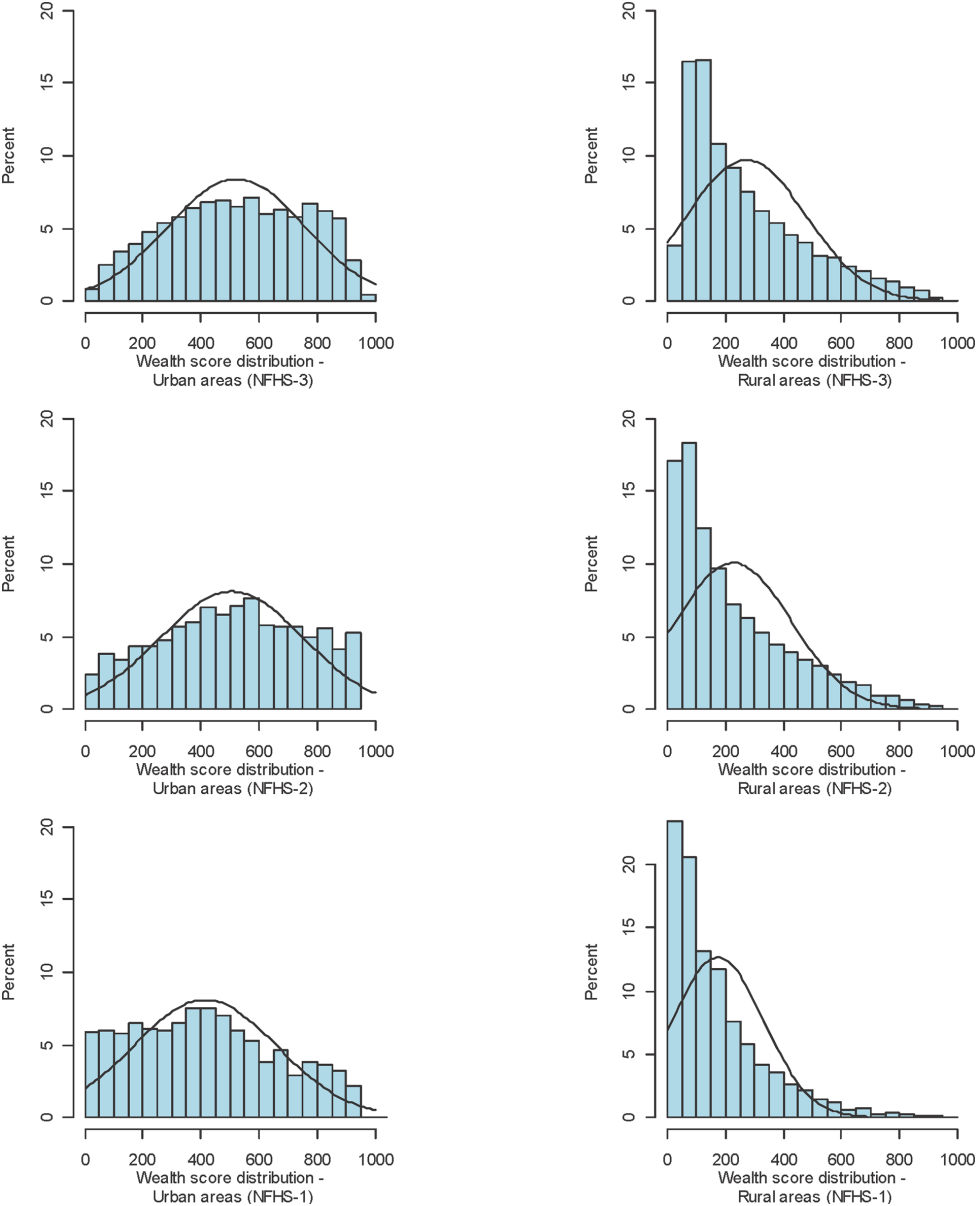
Distribution of wealth scores by urban and rural areas of India across three rounds of the National Family Health Survey (NFHS).

**Figure 5:**
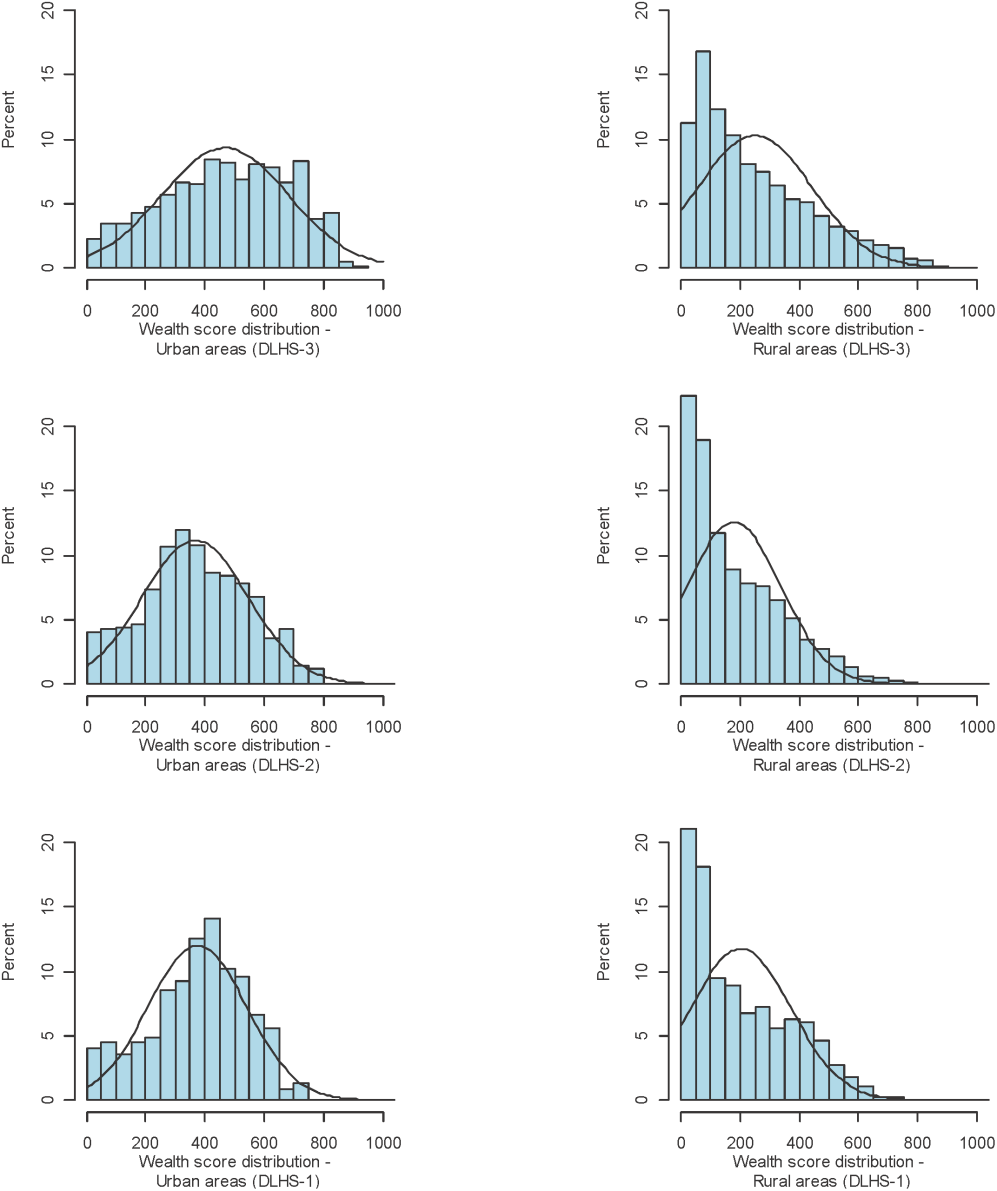
Distribution of wealth scores by urban and rural areas of India across three rounds of the District Level Household Survey (DLHS).

The plotted distributions of household wealth scores by urban and rural areas for each survey round are given in Figures 4 and 5. For the most recent round of the NFHS, in 2005–6, score values for urban households across India ranged from 20 to 955, with a mean score of 547 (standard deviation of 230) and a median score of 552. In rural India, the mean score was 317 (standard deviation of 221) and the median score was 268.

Urban and rural decile cut-off values for India, for the six regions and for the 24 major states are presented by survey round in in Tables 7–12. These wealth score distributions reveal large variability between states, regions, and urban and rural areas. For the most recent NFHS round (2005–6), median scores in urban areas were highest in Delhi (665), followed by Goa (663), Uttaranchal (653), Himachal Pradesh (649), and Punjab (648), with all but Goa located in the North region. The North region’s median score (646) is similar or higher than the seventh decile cut-off values of all other regions. The poorest urban areas were in the states of Tamil Nadu (382) and Andhra Pradesh (394), with median scores that are lower than the third decile cut-off values of 11 other states.

**Table 7:** Decile cut-off values from regional, state and rural/urban distributions of the national wealth index score calculated from the NFHS-3 survey (2005–6) household sample.

			Percentile								
	Region/State		10	20	30	40	50	60	70	80	90
**Rural**	**South**		**75**	**121**	**174**	**234**	**294**	**352**	**419**	**518**	**674**
	AP	Andhra Pradesh	78	136	192	243	301	339	391	455	578
	KA	Karnataka	70	75	123	163	211	272	350	447	582
	KE	Kerala	264	375	463	525	588	665	733	795	885
	TN	Tamil Nadu	70	107	155	203	254	305	374	464	607
	**Central**		**54**	**76**	**100**	**114**	**148**	**193**	**259**	**366**	**534**
	RJ	Rajasthan	54	76	99	115	155	200	273	367	538
	UP	Uttar Pradesh	54	76	92	108	144	192	265	388	557
	CH	Chhattisgarh	54	78	107	131	167	199	254	336	465
	MP	Madhya Pradesh	54	78	102	115	145	181	243	328	487
	**East**		**46**	**54**	**76**	**102**	**130**	**175**	**240**	**336**	**487**
	BH	Bihar	44	54	76	102	124	169	233	320	476
	WB	West Bengal	44	60	90	121	159	205	269	371	518
	JH	Jharkhand	44	54	76	98	114	131	169	230	375
	OR	Orissa	44	54	75	89	114	161	243	356	519
	**Northeast**		**78**	**137**	**197**	**235**	**274**	**323**	**398**	**499**	**638**
	SK	Sikkim	174	264	327	389	444	488	522	588	683
	AR	Arunachal Pradesh	60	97	141	199	259	353	444	540	674
	NA	Nagaland	122	196	249	298	356	416	484	554	661
	MN	Manipur	167	240	302	365	424	474	534	611	704
	MZ	Mizoram	199	293	336	374	422	465	511	603	740
	TR	Tripura	107	182	241	296	352	396	452	509	610
	MG	Meghalaya	76	143	183	235	281	327	385	470	589
	AS	Assam	76	122	182	228	252	290	353	470	630
	**North**		**173**	**253**	**334**	**412**	**496**	**583**	**671**	**767**	**883**
	JM	Jammu and Kashmir	135	205	256	308	370	434	516	603	722
	HP	Himachal Pradesh	225	341	429	501	577	636	722	802	886
	PJ	Punjab	240	341	427	515	597	682	758	848	928
	UC	Uttaranchal	107	184	257	337	425	498	582	677	830
	HR	Haryana	153	221	288	354	425	520	614	727	841
	DL	Delhi	257	350	417	529	608	697	752	848	914
	**West**		**70**	**107**	**163**	**221**	**291**	**368**	**454**	**558**	**704**
	GJ	Gujarat	99	144	200	280	347	412	497	610	762
	MH	Maharashtra	54	99	139	188	256	334	419	519	658
	GO	Goa	168	303	395	496	595	699	785	867	942
	**Rural India**		**54**	**78**	**108**	**155**	**207**	**276**	**359**	**471**	**632**
**Urban**	**South**		**139**	**210**	**292**	**361**	**440**	**511**	**596**	**684**	**776**
	AP	Andhra Pradesh	139	208	271	332	394	461	544	634	745
	KA	Karnataka	144	261	340	418	499	562	627	698	774
	KE	Kerala	288	392	472	532	594	656	709	755	807
	TN	Tamil Nadu	113	174	239	303	382	450	560	666	781
	**Central**		**139**	**223**	**321**	**407**	**485**	**565**	**657**	**748**	**826**
	RJ	Rajasthan	196	283	383	468	534	630	715	795	852
	UP	Uttar Pradesh	109	209	296	388	473	541	636	728	812
	CH	Chhattisgarh	139	202	277	386	472	561	653	738	816
	MP	Madhya Pradesh	135	218	315	391	475	556	644	745	821
	**East**		**135**	**204**	**283**	**377**	**449**	**530**	**608**	**691**	**769**
	BH	Bihar	113	177	240	344	424	494	560	643	757
	WB	West Bengal	144	231	299	384	449	532	615	691	757
	JH	Jharkhand	104	210	323	410	493	577	667	750	816
	OR	Orissa	97	148	229	322	443	537	648	733	795
	**Northeast**		**196**	**279**	**358**	**429**	**485**	**537**	**603**	**684**	**790**
	SK	Sikkim	362	444	476	511	538	588	626	676	713
	AR	Arunachal Pradesh	117	213	305	388	475	537	596	679	774
	NA	Nagaland	235	331	388	449	485	534	590	653	736
	MN	Manipur	235	323	388	442	492	546	608	681	776
	MZ	Mizoram	331	388	434	493	560	611	665	736	793
	TR	Tripura	212	275	327	384	420	499	570	656	736
	MG	Meghalaya	244	323	380	414	461	511	569	642	734
	AS	Assam	170	263	349	429	489	537	608	691	794
	**North**		**231**	**343**	**455**	**560**	**646**	**707**	**764**	**795**	**852**
	JM	Jammu and Kashmir	257	334	410	515	595	681	738	793	852
	HP	Himachal Pradesh	297	424	515	597	649	705	736	774	826
	PJ	Punjab	234	342	459	560	648	712	764	800	852
	UC	Uttaranchal	209	370	475	570	653	710	764	795	852
	HR	Haryana	205	322	405	520	619	679	731	769	826
	DL	Delhi	235	367	488	582	665	724	767	800	852
	**West**		**239**	**344**	**418**	**477**	**537**	**603**	**662**	**722**	**781**
	GJ	Gujarat	261	366	423	475	534	596	658	719	781
	MH	Maharashtra	231	331	410	476	538	603	665	722	781
	GO	Goa	266	389	494	594	663	719	755	786	826
	**Urban India**		**149**	**257**	**334**	**418**	**495**	**570**	**651**	**724**	**795**

**Table 8:** Decile cut-off values from regional, state and rural/urban distributions of the national wealth index score calculated from the NFHS-2 survey (1998–9) household sample.

			Percentile								
	Region/State		10	20	30	40	50	60	70	80	90
**Rural**	**South**		**42**	**83**	**114**	**153**	**201**	**262**	**332**	**420**	**554**
	AP	Andhra Pradesh	21	67	91	132	178	225	288	364	475
	KA	Karnataka	42	70	91	132	160	215	287	382	529
	KE	Kerala	148	212	258	307	374	420	498	586	746
	TN	Tamil Nadu	44	86	113	139	183	238	310	405	545
	**Central**		**21**	**44**	**67**	**88**	**112**	**153**	**204**	**298**	**459**
	RJ	Rajasthan	21	44	83	107	137	183	252	364	528
	UP	Uttar Pradesh	21	42	65	84	107	134	194	284	451
	CH	Chhattisgarh	NA	NA	NA	NA	NA	NA	NA	NA	NA
	MP	Madhya Pradesh	42	63	70	93	116	156	202	284	443
	**East**		**21**	**42**	**63**	**84**	**104**	**132**	**192**	**275**	**434**
	BH	Bihar	21	42	63	83	91	132	191	258	400
	WB	West Bengal	NA	NA	NA	NA	NA	NA	NA	NA	NA
	JH	Jharkhand	NA	NA	NA	NA	NA	NA	NA	NA	NA
	OR	Orissa	21	42	63	67	88	130	183	286	475
	**Northeast**		**44**	**88**	**136**	**191**	**216**	**258**	**306**	**390**	**521**
	SK	Sikkim	133	219	265	319	379	426	495	554	661
	AR	Arunachal Pradesh	70	114	162	208	245	299	385	491	603
	NA	Nagaland	88	150	198	240	286	338	391	445	557
	MN	Manipur	88	153	202	247	287	335	389	475	607
	MZ	Mizoram	178	275	303	326	368	393	416	460	512
	TR	Tripura	70	132	180	216	260	290	353	410	494
	MG	Meghalaya	42	65	91	136	193	235	265	320	414
	AS	Assam	42	84	127	170	212	237	281	350	502
	**North**		**137**	**215**	**288**	**368**	**443**	**514**	**582**	**648**	**748**
	JM	Jammu and Kashmir	108	172	225	280	332	393	471	548	648
	HP	Himachal Pradesh	168	258	348	420	483	546	603	649	756
	PJ	Punjab	160	267	360	440	509	571	628	699	790
	UC	Uttaranchal	NA	NA	NA	NA	NA	NA	NA	NA	NA
	HR	Haryana	91	170	241	300	374	452	520	590	690
	DL	Delhi	295	410	469	536	586	631	707	759	864
	**West**		**42**	**70**	**109**	**137**	**186**	**260**	**335**	**440**	**589**
	GJ	Gujarat	42	87	116	176	240	306	390	471	600
	MH	Maharashtra	42	67	93	132	171	227	303	406	572
	GO	Goa	159	263	351	410	479	571	658	767	891
	**Rural India**		**42**	**63**	**86**	**111**	**153**	**206**	**282**	**387**	**543**
**Urban**	**South**		**115**	**203**	**272**	**336**	**399**	**464**	**537**	**645**	**761**
	AP	Andhra Pradesh	113	182	261	314	380	445	515	624	739
	KA	Karnataka	115	229	317	399	464	520	603	716	788
	KE	Kerala	227	317	361	405	470	519	603	698	768
	TN	Tamil Nadu	113	178	230	288	338	401	476	581	721
	**Central**		**113**	**203**	**289**	**369**	**442**	**509**	**563**	**655**	**765**
	RJ	Rajasthan	134	215	300	383	448	516	586	676	786
	UP	Uttar Pradesh	125	227	306	384	445	511	578	655	763
	CH	Chhattisgarh	NA	NA	NA	NA	NA	NA	NA	NA	NA
	MP	Madhya Pradesh	88	165	247	333	420	489	553	650	761
	**East**		**94**	**164**	**245**	**312**	**399**	**464**	**533**	**624**	**724**
	BH	Bihar	88	150	227	307	380	451	514	586	694
	WB	West Bengal	NA	NA	NA	NA	NA	NA	NA	NA	NA
	JH	Jharkhand	NA	NA	NA	NA	NA	NA	NA	NA	NA
	OR	Orissa	50	75	140	242	332	421	513	619	761
	**Northeast**		**177**	**277**	**337**	**397**	**445**	**495**	**558**	**631**	**743**
	SK	Sikkim	265	349	380	399	449	514	562	612	721
	AR	Arunachal Pradesh	140	240	290	355	420	464	489	560	677
	NA	Nagaland	226	330	370	399	445	489	536	590	714
	MN	Manipur	201	266	315	355	388	442	495	567	667
	MZ	Mizoram	330	355	377	402	442	491	560	653	743
	TR	Tripura	152	246	334	380	419	464	541	628	703
	MG	Meghalaya	203	290	334	380	405	445	500	587	696
	AS	Assam	139	241	324	439	489	536	586	675	790
	**North**		**293**	**414**	**489**	**558**	**630**	**697**	**763**	**807**	**857**
	JM	Jammu and Kashmir	253	330	370	417	489	539	620	711	793
	HP	Himachal Pradesh	332	420	467	525	586	651	703	761	807
	PJ	Punjab	414	486	555	619	694	760	793	832	857
	UC	Uttaranchal	NA	NA	NA	NA	NA	NA	NA	NA	NA
	HR	Haryana	243	345	444	514	605	672	757	807	857
	DL	Delhi	290	407	492	561	644	711	765	807	857
	**West**		**155**	**246**	**317**	**395**	**450**	**514**	**597**	**676**	**760**
	GJ	Gujarat	153	262	370	439	486	556	642	714	788
	MH	Maharashtra	157	230	311	376	432	493	565	650	735
	GO	Goa	202	312	405	496	570	650	721	786	832
	**Urban India**		**125**	**222**	**299**	**376**	**445**	**511**	**584**	**677**	**782**

**Table 9:** Decile cut-off values from regional, state and rural/urban distributions of the national wealth index score calculated from the NFHS-1 survey (1992–3) household sample.

			Percentile								
	Region/State		10	20	30	40	50	60	70	80	90
**Rural**	**South**		**22**	**53**	**82**	**104**	**135**	**166**	**219**	**304**	**454**
	AP	Andhra Pradesh	22	44	66	84	106	137	178	250	346
	KA	Karnataka	22	44	82	104	128	159	191	241	368
	KE	Kerala	88	119	168	219	287	349	445	561	721
	TN	Tamil Nadu	22	53	75	88	113	148	197	283	431
	**Central**		**22**	**44**	**66**	**84**	**104**	**128**	**157**	**213**	**356**
	RJ	Rajasthan	22	44	53	82	104	135	181	262	401
	UP	Uttar Pradesh	22	44	53	82	88	119	150	193	318
	CH	Chhattisgarh	NA	NA	NA	NA	NA	NA	NA	NA	NA
	MP	Madhya Pradesh	22	44	82	88	113	135	175	241	388
	**East**		**22**	**44**	**44**	**66**	**84**	**106**	**137**	**169**	**284**
	BH	Bihar	22	44	44	66	75	88	119	150	241
	WB	West Bengal	NA	NA	NA	NA	NA	NA	NA	NA	NA
	JH	Jharkhand	NA	NA	NA	NA	NA	NA	NA	NA	NA
	OR	Orissa	22	44	44	66	75	104	137	181	315
	**Northeast**		**22**	**44**	**66**	**88**	**104**	**128**	**166**	**227**	**396**
	SK	Sikkim	NA	NA	NA	NA	NA	NA	NA	NA	NA
	AR	Arunachal Pradesh	44	66	82	104	129	166	210	308	472
	NA	Nagaland	75	116	140.5	166	210	256	296	366	487
	MN	Manipur	66	88	113	129	157	188	222	303	488
	MZ	Mizoram	53	75	128	157	182	220	253	304	422
	TR	Tripura	22	53	75	88	115	146	181	250	388
	MG	Meghalaya	44	66	75	97	119	148	197	272	432
	AS	Assam	22	44	66	75	97	119	150	196	370
	**North**		**104**	**178**	**224**	**284**	**326**	**388**	**455**	**535**	**700**
	JM	Jammu and Kashmir	82	135	193	253	325	387	472	552	691
	HP	Himachal Pradesh	104	135	179	222	273	326	391	495	585
	PJ	Punjab	135	200	263	316	366	431	504	619	824
	UC	Uttaranchal	NA	NA	NA	NA	NA	NA	NA	NA	NA
	HR	Haryana	82	140	200	262	306	347	409	494	598
	DL	Delhi	204	262	308	396	451	517	586	725	993
	**West**		**22**	**53**	**82**	**106**	**135**	**175**	**231**	**315**	**463**
	GJ	Gujarat	22	53	84	113	144	206	271	356	527
	MH	Maharashtra	22	53	82	104	135	166	200	284	419
	GO	Goa	104	144	188	251	326	431	536	690	901
	**Rural India**		**22**	**44**	**66**	**88**	**106**	**144**	**188**	**266**	**423**
**Urban**	**South**		**97**	**152**	**224**	**299**	**353**	**412**	**473**	**541**	**627**
	AP	Andhra Pradesh	77	155	247	316	374	428	478	547	645
	KA	Karnataka	97	151	203	263	339	420	474	548	641
	KE	Kerala	122	195	249	303	366	417	478	556	641
	TN	Tamil Nadu	77	149	212	293	337	391	451	504	600
	**Central**		**96**	**170**	**247**	**320**	**381**	**450**	**510**	**600**	**696**
	RJ	Rajasthan	97	166	251	326	391	452	504	600	696
	UP	Uttar Pradesh	77	151	224	300	365	433	510	611	713
	CH	Chhattisgarh	NA	NA	NA	NA	NA	NA	NA	NA	NA
	MP	Madhya Pradesh	121	205	291	351	412	464	523	596	677
	**East**		**69**	**106**	**180**	**263**	**347**	**420**	**475**	**553**	**644**
	BH	Bihar	52	104	177	297	397	451	525	600	692
	WB	West Bengal	NA	NA	NA	NA	NA	NA	NA	NA	NA
	JH	Jharkhand	NA	NA	NA	NA	NA	NA	NA	NA	NA
	OR	Orissa	46	97	146	220	293	359	426	502	620
	**Northeast**		**81**	**150**	**209**	**278**	**351**	**412**	**482**	**552**	**648**
	SK	Sikkim	NA	NA	NA	NA	NA	NA	NA	NA	NA
	AR	Arunachal Pradesh	75	120	149	195	255	355	428	487	570
	NA	Nagaland	173	248	309	365	420	457	507	527	598
	MN	Manipur	92	127	178	204	255	305	407	499	573
	MZ	Mizoram	149	201	227	262	296	331	384.5	441	574
	TR	Tripura	148	203	255	305	351	388	450	517	560
	MG	Meghalaya	174	205	262	302	341	400	430	475	556
	AS	Assam	69	123	203	284	381	451	525	600	696
	**North**		**199**	**282**	**350**	**404**	**465**	**527**	**604**	**671**	**738**
	JM	Jammu and Kashmir	277	345	404	448	504	567	626	677	721
	HP	Himachal Pradesh	227	310	366	408	469	510	577	638	700
	PJ	Punjab	224	301	369	420	481	541	619	677	721
	UC	Uttaranchal	NA	NA	NA	NA	NA	NA	NA	NA	NA
	HR	Haryana	193	275	335	397	439	500	571	638	715
	DL	Delhi	180	270	345	404	464	540	625	686	738
	**West**		**106**	**180**	**253**	**307**	**365**	**420**	**476**	**554**	**646**
	GJ	Gujarat	97	180	253	305	353	405	458	548	654
	MH	Maharashtra	106	180	253	307	370	422	478	554	642
	GO	Goa	151	247	321	386	444	524	611	667	713
	**Urban India**		**97**	**166**	**239**	**307**	**374**	**428**	**493**	**573**	**671**

**Table 10:** Decile cut-off values from regional, state and rural/urban distributions of the national wealth index score calculated from the DLHS-3 survey (2007–8) household sample.

			Percentile								
	Region/State		10	20	30	40	50	60	70	80	90
**Rural**	**South**		**74**	**106**	**153**	**198**	**251**	**299**	**362**	**438**	**560**
	AP	Andhra Pradesh	74	96	151	191	232	271	315	366	449
	KA	Karnataka	74	74	116	141	189	235	291	366	465
	KE	Kerala	192	276	347	407	460	509	578	648	719
	LK	Lakshadweep	312	394	442	496	556	593	628	670	731
	PY	Pondicherry	77	143	191	235	277	319	366	449	560
	TN	Tamil Nadu	74	99	153	191	234	273	319	381	482
	AN	Andaman and Nicobar Islands	96	151	219	295	360	425	489	564	665
	**Central**		**20**	**42**	**74**	**94**	**125**	**165**	**225**	**319**	**474**
	RJ	Rajasthan	20	61	83	116	159	215	289	387	527
	UP	Uttar Pradesh	20	40	61	81	105	146	210	314	487
	CG	Chhattisgarh	219	370	467	528	592	620	646	707	762
	MP	Madhya Pradesh	40	74	84	115	135	172	219	298	438
	**East**		**40**	**61**	**74**	**83**	**105**	**138**	**182**	**253**	**386**
	BH	Bihar	61	61	81	103	118	141	182	242	365
	WB	West Bengal	20	42	64	84	121	170	227	312	430
	JH	Jharkhand	162	283	379	449	509	567	620	666	741
	OR	Orissa	20	40	42	62	77	100	161	267	426
	**Northeast**		**84**	**141**	**191**	**224**	**265**	**316**	**363**	**426**	**510**
	SK	Sikkim	179	239	287	335	361	403	427	462	517
	AR	Arunachal Pradesh	131	191	233	272	304	344	390	443	516
	NA	Nagaland	NA	NA	NA	NA	NA	NA	NA	NA	NA
	MN	Manipur	137	183	228	262	296	340	385	447	529
	MZ	Mizoram	137	204	264	299	321	343	385	430	520
	TR	Tripura	42	99	157	200	254	314	356	408	489
	MG	Meghalaya	80	122	159	198	231	260	296	354	427
	AS	Assam	62	104	157	181	220	252	313	403	514
	**North**		**150**	**219**	**281**	**341**	**402**	**466**	**536**	**617**	**707**
	JM	Jammu and Kashmir	135	192	242	286	331	381	441	513	615
	HP	Himachal Pradesh	205	286	345	399	448	498	550	607	689
	PJ	Punjab	218	312	392	472	550	616	665	707	762
	UC	Uttaranchal	123	179	229	283	339	390	449	512	599
	HR	Haryana	121	178	241	306	376	448	532	624	708
	CH	Chandigarh	40	62	74	96	118	153	193	255	372
	DL	Delhi	163	265	336	409	476	556	619	687	736
	**West**		**61**	**105**	**151**	**197**	**254**	**314**	**379**	**454**	**560**
	GJ	Gujarat	83	135	175	223	274	325	388	465	560
	DD	Daman & Diu	232	286	328	377	412	465	509	580	644
	DN	Dadra & Nagar Haveli	94	137	175	219	252	292	346	409	502
	MH	Maharashtra	40	74	117	160	220	283	348	427	537
	GO	Goa	191	262	330	390	472	542	624	697	749
	**Rural India**		**42**	**74**	**105**	**145**	**198**	**256**	**328**	**420**	**547**
**Urban**	**South**		**156**	**249**	**314**	**375**	**433**	**495**	**560**	**642**	**718**
	AP	Andhra Pradesh	202	276	336	392	447	509	577	646	721
	KA	Karnataka	119	202	277	344	407	461	521	594	678
	KE	Kerala	300	399	469	525	582	643	673	729	773
	LK	Lakshadweep	379	475.5	553	604	654	688.5	715	746.5	778
	PY	Pondicherry	214	295	359	426	493	562	626	673	731
	TN	Tamil Nadu	134	213	276	326	381	437	498	573	666
	AN	Andaman and Nicobar Islands	311	396	467	509	553	584	636	666	719
	**Central**		**94**	**188**	**279**	**363**	**443**	**516**	**595**	**669**	**749**
	RJ	Rajasthan	173	282	370	450	526	599	665	729	802
	UP	Uttar Pradesh	62	134	219	310	399	484	558	643	729
	CG	Chhattisgarh	399	529	616	666	708	729	781	804	822
	MP	Madhya Pradesh	119	217	300	374	446	515	583	666	747
	**East**		**103**	**192**	**290**	**376**	**447**	**509**	**578**	**646**	**721**
	BH	Bihar	81	118	182	271	367	450	529	599	687
	WB	West Bengal	134	242	324	390	445	494	569	640	702
	JH	Jharkhand	162	283	379	449	509	567	620	666	741
	OR	Orissa	74	156	255	352	433	513	580	652	735
	**Northeast**		**252**	**341**	**400**	**443**	**487**	**528**	**578**	**644**	**718**
	SK	Sikkim	343	405	429	448	489	515	535	584	636
	AR	Arunachal Pradesh	274	343	390	428	466	497	529	584	666
	NA	Nagaland	NA	NA	NA	NA	NA	NA	NA	NA	NA
	MN	Manipur	226	313	381	425	471	514	560	602	672
	MZ	Mizoram	299	343	401	427	478	530	583	647	722
	TR	Tripura	257	336	385	427	487	538	592	657	729
	MG	Meghalaya	199	277	341	383	427	465	506	560	644
	AS	Assam	182	316	406	467	500	550	583	649	729
	**North**		**264**	**365**	**445**	**526**	**593**	**646**	**707**	**733**	**800**
	JM	Jammu and Kashmir	302	370	419	467	526	595	667	711	773
	HP	Himachal Pradesh	321	429	486	538	586	636	684	729	782
	PJ	Punjab	289	419	528	600	647	707	727	776	804
	UC	Uttaranchal	286	374	443	500	560	618	666	729	782
	HR	Haryana	227	326	423	509	580	644	707	729	802
	CH	Chandigarh	118	195	266	345	425	502	580	663	729
	DL	Delhi	249	343	429	509	576	638	687	729	782
	**West**		**235**	**322**	**387**	**445**	**493**	**553**	**622**	**666**	**729**
	GJ	Gujarat	261	343	403	456	508	564	633	666	739
	DD	Daman & Diu	381	465	500	557	606	644	675	719	741
	DN	Dadra & Nagar Haveli	276	340	366	410	467	489	553	624	649
	MH	Maharashtra	204	299	363	423	473	528	575	646	719
	GO	Goa	292	410	498	573	625	670	709	741	782
	**Urban India**		**158**	**265**	**347**	**418**	**482**	**547**	**614**	**673**	**741**

**Table 11:** Decile cut-off values from regional, state and rural/urban distributions of the national wealth index score calculated from the DLHS-2 survey (2002–4) household sample.

			Percentile								
	Region/State		10	20	30	40	50	60	70	80	90
**Rural**	**South**		**26**	**73**	**101**	**146**	**182**	**229**	**283**	**337**	**431**
	AP	Andhra Pradesh	0	73	78	146	172	221	255	285	360
	KA	Karnataka	26	73	73	101	129	179	229	290	391
	KE	Kerala	52	80	136	202	254	309	363	429	504
	LK	Lakshadweep	200	266	315	341	374	401	429	457	496
	PY	Pondicherry	73	146	200	229	283	311	363	428	520
	TN	Tamil Nadu	26	73	101	155	202	254	287	337	421
	AN	Andaman and Nicobar Islands	73	146	202	257	309	337	412	459	532
	**Central**		**0**	**26**	**52**	**73**	**99**	**134**	**182**	**256**	**363**
	RJ	Rajasthan	0	0	52	73	127	172	229	308	412
	UP	Uttar Pradesh	0	26	26	56	82	108	157	238	363
	CG	Chhattisgarh	184	256	293	311	339	363	403	480	595
	MP	Madhya Pradesh	0	28	73	82	101	153	200	256	348
	**East**		**0**	**0**	**26**	**52**	**56**	**82**	**129**	**182**	**301**
	BH	Bihar	0	0	26	28	56	82	106	153	246
	WB	West Bengal	0	26	28	54	80	106	155	228	313
	JH	Jharkhand	78	172	255	311	363	414	482	557	628
	OR	Orissa	0	0	26	52	56	83	146	228	335
	**Northeast**		**0**	**52**	**73**	**99**	**129**	**162**	**212**	**287**	**386**
	SK	Sikkim	73	101	125	153	184	212	264	323	386
	AR	Arunachal Pradesh	0	52	73	99	129	181	235	307	391
	NA	Nagaland	0	56	73	125	129	181	256	337	433
	MN	Manipur	56	101	129	153	181	212	280	337	454
	MZ	Mizoram	28	73	101	129	153	181	211	256	355
	TR	Tripura	28	73	106	153	202	254	285	335	391
	MG	Meghalaya	0	0	28	52	73	101	125	163	236
	AS	Assam	0	26	28	56	80	106	147	252	363
	**North**		**73**	**146**	**200**	**247**	**284**	**330**	**382**	**433**	**523**
	JM	Jammu and Kashmir	73	125	157	208	254	281	309	356	412
	HP	Himachal Pradesh	101	153	208	275	311	360	410	450	524
	PJ	Punjab	172	229	255	311	355	386	438	506	571
	UC	Uttaranchal	0	52	78	127	174	238	303	367	459
	HR	Haryana	82	172	224	255	304	337	386	438	529
	CH	Chandigarh	0	26	54	78	99	127	155	224	311
	DL	Delhi	200	229	285	311	337	388	438	503	623
	**West**		**26**	**73**	**101**	**146**	**174**	**228**	**281**	**335**	**426**
	GJ	Gujarat	26	73	127	146	198	228	268	321	411
	DD	Daman & Diu	129	172	202	229	281	311	348	402	477
	DN	Dadra & Nagar Haveli	73	73	125	146	198	230	280	315	405
	MH	Maharashtra	0	56	73	127	156	208	262	313	404
	GO	Goa	125	198	273	323	376	431	492	548	623
	**Rural India**		**0**	**28**	**73**	**99**	**134**	**182**	**252**	**311**	**410**
**Urban**	**South**		**146**	**228**	**280**	**311**	**355**	**401**	**456**	**522**	**576**
	AP	Andhra Pradesh	146	228	255	285	313	365	413	496	557
	KA	Karnataka	129	212	275	311	363	414	470	540	609
	KE	Kerala	125	202	265	311	363	410	459	522	597
	LK	Lakshadweep	226	281	323	369	401	454	480	548	567
	PY	Pondicherry	174	255	307	351	402	456	513	550	623
	TN	Tamil Nadu	146	228	281	309	337	376	438	501	571
	AN	Andaman and Nicobar Islands	229	307	337	398	431	466	506	550	597
	**Central**		**99**	**200**	**255**	**311**	**358**	**404**	**466**	**534**	**623**
	RJ	Rajasthan	146	229	285	341	386	450	522	573	651
	UP	Uttar Pradesh	73	156	229	285	335	386	438	508	609
	CG	Chhattisgarh	224	311	374	438	529	576	625	651	736
	MP	Madhya Pradesh	146	228	277	311	360	410	468	543	623
	**East**		**54**	**146**	**228**	**283**	**323**	**376**	**433**	**501**	**578**
	BH	Bihar	26	78	150	230	307	358	414	482	578
	WB	West Bengal	82	200	254	285	313	363	403	459	532
	JH	Jharkhand	78	172	255	311	363	414	482	557	628
	OR	Orissa	56	172	255	307	335	391	451	524	599
	**Northeast**		**125**	**200**	**255**	**306**	**348**	**402**	**459**	**524**	**599**
	SK	Sikkim	146	212	257	285	334	358	386	449	497
	AR	Arunachal Pradesh	101	156	212	264	309	358	407	470	550
	NA	Nagaland	181	240	292	358	406	458	488	550	593
	MN	Manipur	108	155	207	254	298	337	391	456	557
	MZ	Mizoram	146	204	256	311	369	433	498	550	625
	TR	Tripura	172	230	285	313	363	405	454	496	576
	MG	Meghalaya	108	157	212	250	298	334	386	445	498
	AS	Assam	56	181	280	311	363	412	480	534	619
	**North**		**229**	**283**	**330**	**365**	**412**	**461**	**529**	**573**	**645**
	JM	Jammu and Kashmir	208	281	296	337	371	412	456	531	625
	HP	Himachal Pradesh	254	329	383	438	466	531	560	623	651
	PJ	Punjab	247	307	358	386	454	515	571	619	656
	UC	Uttaranchal	172	257	311	367	412	461	508	569	645
	HR	Haryana	229	285	339	386	424	477	534	597	651
	CH	Chandigarh	99	172	255	307	339	402	477	548	623
	DL	Delhi	229	281	307	339	382	414	479	545	623
	**West**		**200**	**255**	**285**	**335**	**376**	**423**	**470**	**529**	**597**
	GJ	Gujarat	200	255	285	321	374	414	480	529	597
	DD	Daman & Diu	202	281	311	349	402	451	494	524	596
	DN	Dadra & Nagar Haveli	230	285	321	379	428	496	524	571	633
	MH	Maharashtra	184	255	285	337	376	412	459	522	581
	GO	Goa	157	273	309	365	423	478	545	597	659
	**Urban India**		**129**	**224**	**281**	**311**	**363**	**409**	**466**	**532**	**623**

**Table 12:** Decile cut-off values from regional, state and rural/urban distributions of the national wealth index score calculated from the DLHS-1 survey (1998–9) household sample.

			Percentile								
	Region/State		10	20	30	40	50	60	70	80	90
**Rural**	**South**		**0**	**65**	**97**	**140**	**200**	**258**	**316**	**378**	**443**
	AP	Andhra Pradesh	0	0	78	97	166	228	288	346	411
	KA	Karnataka	30	78	90	138	168	200	258	323	413
	KE	Kerala	60	95	138	200	260	316	378	411	488
	LK	Lakshadweep	261	288	316	323	353	403	431	463	498
	PY	Pondicherry	NA	NA	NA	NA	NA	NA	NA	NA	NA
	TN	Tamil Nadu	30	78	122	173	228	288	323	383	446
	AN	Andaman and Nicobar Islands	97	168	230	267	321	371	408	458	523
	**Central**		**0**	**35**	**78**	**97**	**140**	**198**	**263**	**353**	**458**
	RJ	Rajasthan	0	60	95	166	226	288	348	413	488
	UP	Uttar Pradesh	30	35	65	95	115	166	237	335	443
	CG	Chhattisgarh	NA	NA	NA	NA	NA	NA	NA	NA	NA
	MP	Madhya Pradesh	0	60	78	113	143	201	261	341	433
	**East**		**0**	**0**	**35**	**60**	**92**	**97**	**157**	**235**	**353**
	BH	Bihar	0	0	35	60	90	97	152	187	323
	WB	West Bengal	0	30	35	65	95	122	166	261	362
	JH	Jharkhand	NA	NA	NA	NA	NA	NA	NA	NA	NA
	OR	Orissa	0	0	35	60	95	125	173	291	413
	**Northeast**		**0**	**30**	**60**	**78**	**95**	**138**	**183**	**261**	**370**
	SK	Sikkim	30	78	78	108	138	168	200	230	290
	AR	Arunachal Pradesh	0	0	0	60	78	78	138	168	290
	NA	Nagaland	0	60	80	122	168	228	291	370	441
	MN	Manipur	0	60	62	95	138	157	200	265	353
	MZ	Mizoram	78	108	138	168	200	218	280	310	383
	TR	Tripura	0	30	60	108	166	256	323	378	413
	MG	Meghalaya	0	30	60	62	92	140	170	230	308
	AS	Assam	0	0	35	60	65	97	157	228	381
	**North**		**140**	**201**	**260**	**311**	**368**	**401**	**446**	**488**	**526**
	JM	Jammu and Kashmir	140	168	226	258	310	355	398	458	493
	HP	Himachal Pradesh	108	170	230	306	368	398	458	458	493
	PJ	Punjab	201	281	341	373	431	436	493	526	588
	UC	Uttaranchal	NA	NA	NA	NA	NA	NA	NA	NA	NA
	HR	Haryana	95	201	256	306	351	398	431	493	528
	CH	Chandigarh	NA	NA	NA	NA	NA	NA	NA	NA	NA
	DL	Delhi	NA	NA	NA	NA	NA	NA	NA	NA	NA
	**West**		**35**	**78**	**138**	**173**	**226**	**263**	**321**	**381**	**458**
	GJ	Gujarat	60	113	170	226	256	288	323	381	446
	DD	Daman & Diu	166	201	231	263	306	326	358	413	463
	DN	Dadra & Nagar Haveli	65	78	113	166	201	258	318	381	458
	MH	Maharashtra	30	78	108	138	185	235	316	371	446
	GO	Goa	138	226	286	323	370.5	411	458	508	588
	**Rural India**		**0**	**35**	**78**	**108**	**157**	**223**	**290**	**370**	**458**
**Urban**	**South**		**122**	**228**	**291**	**348**	**378**	**413**	**448**	**503**	**556**
	AP	Andhra Pradesh	78	173	256	316	351	383	441	493	538
	KA	Karnataka	78	168	256	316	356	411	448	508	583
	KE	Kerala	110	200	286	316	351	383	426	488	538
	LK	Lakshadweep	283	323	371	413	443	493	508	558	588
	PY	Pondicherry	108	226	288	323	378	383	413	458	508
	TN	Tamil Nadu	196	288	325	378	411	418	476	508	583
	AN	Andaman and Nicobar Islands	NA	NA	NA	NA	NA	NA	NA	NA	NA
	**Central**		**143**	**256**	**316**	**366**	**413**	**458**	**493**	**528**	**618**
	RJ	Rajasthan	226	291	351	403	443	488	493	526	591
	UP	Uttar Pradesh	95	201	286	346	396	433	493	526	618
	CG	Chhattisgarh	NA	NA	NA	NA	NA	NA	NA	NA	NA
	MP	Madhya Pradesh	166	261	321	368	413	458	493	538	618
	**East**		**60**	**157**	**261**	**323**	**373**	**413**	**443**	**508**	**583**
	BH	Bihar	60	97	201	306	353	413	488	523	618
	WB	West Bengal	95	226	288	323	353	396	413	458	523
	JH	Jharkhand	NA	NA	NA	NA	NA	NA	NA	NA	NA
	OR	Orissa	60	166	291	351	401	443	500	538	618
	**Northeast**		**90**	**140**	**200**	**260**	**310**	**351**	**408**	**465**	**553**
	SK	Sikkim	170	226	228	290	316	336	370	411	545
	AR	Arunachal Pradesh	78	108	140	198	238	306	348	408	478
	NA	Nagaland	90	166	226	280	310	338	378	413	495
	MN	Manipur	95	138	173	203	235	290	353	413	523
	MZ	Mizoram	138	170	200	250	280	325	396	458	553
	TR	Tripura	30	108	196	288	318	351	383	443	488
	MG	Meghalaya	138	200	260	290	290	320	370	408	488
	AS	Assam	95	203	321	381	443	488	523	583	618
	**North**		**286**	**353**	**413**	**441**	**491**	**523**	**553**	**588**	**618**
	JM	Jammu and Kashmir	NA	NA	NA	NA	NA	NA	NA	NA	NA
	HP	Himachal Pradesh	336	398	458	461	493	523	583	583	648
	PJ	Punjab	311	381	431	461	493	526	556	588	621
	UC	Uttaranchal	261	341	396	431	463	493	526	588	618
	HR	Haryana	NA	NA	NA	NA	NA	NA	NA	NA	NA
	CH	Chandigarh	NA	NA	NA	NA	NA	NA	NA	NA	NA
	DL	Delhi	NA	NA	NA	NA	NA	NA	NA	NA	NA
	**West**		**218**	**288**	**321**	**378**	**396**	**413**	**458**	**503**	**556**
	GJ	Gujarat	226	291	351	381	413	446	493	528	588
	DD	Daman & Diu	265	318	351	383	413	443	493	508	568
	DN	Dadra & Nagar Haveli	306	351	381	411	441	503	538	583	618
	MH	Maharashtra	196	268	316	351	378	408	431	476	523
	GO	Goa	226	316	378	413	458	503	528	583	618
	**Urban India**		**127**	**228**	**306**	**351**	**386**	**431**	**473**	**523**	**588**

Rural wealth score gaps are even larger than those observed in urban areas. Median scores in rural areas for 2005–6 (NFHS) were highest in Delhi (608), followed by Punjab (597), Goa (595) and Kerala (588), with very low median scores in the Eastern states of Jharkhand (114), Orissa (114), Bihar (124) and the Central states of Madhya Pradesh (145) and Uttar Pradesh (144). The median scores of Jharkhand and Orissa are lower than the first decile cut-off values for 11 other states, and lower than the second decile cut-off values for 17 other states. The region with the richest rural areas is the North, with a median score of 496, followed from a considerable distance by the West region with a median score of 291.

## Discussion

PCA has been previously evaluated and used for the development of wealth scores based on household asset data, including the NFHS itself [17], [18], [20]. The present analysis adds a careful consideration of the sub-national variation in wealth and the differences in wealth index scores and components by rural and urban areas. As has been shown previously in Brazil [15], there is large variability in sub-national distributions of scores and there are many benefits in taking the variation into account. For example, it allows for within-sample economic classification, and for comparisons across geographical and urban/rural distributions.

Unlike the original PCA-based wealth score that is made available with the NFHS datasets, which has a common number of items and scores for a national distribution, the indices were constructed so as to allow for the identification of regional and state-level decile cut-off points for urban and rural households separately. This enables the scores to be used for comparisons at different levels of aggregation, and the importance of local distribution cut-off points is illustrated by the state-level examples in Figures 1 and 2. In addition, changes in the index components and in their coefficients over time – from 1992–3 (NFHS-1) to 2005–06 (NFHS-3) for example – illustrate the need to revise indices periodically.

The items that we included in the national wealth indices are relatively simple to measure in population surveys, and are limited 15 or fewer assets, thereby limiting the time needed to collect wealth data during a household interview. In addition, future analyses of the NFHS datasets can take direct advantage of the wealth indices and the sub-national score distributions presented here. Other variables that were available in the NFHS surveys were not included because they did not contribute importantly to the score and/or were not required to improve the distribution. Radio and bed are two examples of items that had a lower loading (less than 0.2) and were kept in the calculations to improve the distribution of the score by avoiding accumulation of households in a specific decile (a function of having too many households reporting ownership of a very limited number of items, especially in rural areas).

The resulting scores are valid indicators of wealth that correlate well with health outcomes, as seen by the variation in the mean height-for-age scores (Figure 1) and in stunting prevalence across the wealth score quintiles (Figure 2). The proportion of the total variability explained by the first component of the urban (ranging from 39.5% and 41.4%) and rural scores (ranging from 33.6% to 33.8%) can be considered high given the size of India’s population and its income inequality (Gini index: 33.9 in 2010 [31]).

In summary, we constructed valid asset-based wealth indices from six nationally representative surveys of households in India conducted between 1992–3 and 2007–8, and we present the regional and state-level distributions of these wealth scores for urban and rural areas separately. These scores can be used for analyses within the source surveys to understand differences within and across geographical levels, and for ecological analyses that combine the source surveys with other datasets. In addition to the wide variety of scenarios in which these indices can be currently applied, they are also based on data that could be collected relatively easily in future studies.
